# Ecotype Evolution in *Glossina palpalis* Subspecies, Major Vectors of Sleeping Sickness

**DOI:** 10.1371/journal.pntd.0003497

**Published:** 2015-03-16

**Authors:** Thierry De Meeûs, Jérémy Bouyer, Sophie Ravel, Philippe Solano

**Affiliations:** 1 IRD (INTERTRYP), UMR 177 IRD-CIRAD, Centre International de Recherche-Développement sur l'Elevage en zone Subhumide (CIRDES), Bobo-Dioulasso, Burkina-Faso; 2 IRD (INTERTRYP), UMR177 IRD-CIRAD, TA A-17/G, Campus International de Baillarguet, Montpellier, France; 3 Unité Mixte de Recherche Contrôle des Maladies Animales Exotiques et Emergentes, Centre de Coopération Internationale en Recherche Agronomique pour le Développement (CIRAD), Montpellier, France; 4 Unité Mixte de Recherche 1309 Contrôle des Maladies Animales Exotiques et Emergentes, Institut national de la recherche agronomique (INRA), Montpellier, France; 5 Institut Sénégalais de Recherches Agricoles, Laboratoire National d'Elevage et de Recherches Vétérinaires, Dakar—Hann, Sénégal; Yale University, UNITED STATES

## Abstract

**Background:**

The role of environmental factors in driving adaptive trajectories of living organisms is still being debated. This is even more important to understand when dealing with important neglected diseases and their vectors.

**Methodology/Principal Findings:**

In this paper, we analysed genetic divergence, computed from seven microsatellite loci, of 614 tsetse flies (*Glossina palpalis gambiensis* and *Glossina palpalis palpalis*, major vectors of animal and human trypanosomes) from 28 sites of West and Central Africa. We found that the two subspecies are so divergent that they deserve the species status. Controlling for geographic and time distances that separate these samples, which have a significant effect, we found that *G*. *p*. *gambiensis* from different landscapes (Niayes of Senegal, savannah and coastal environments) were significantly genetically different and thus represent different ecotypes or subspecies. We also confirm that *G*. *p*. *palpalis* from Ivory Coast, Cameroon and DRC are strongly divergent.

**Conclusions/Significance:**

These results provide an opportunity to examine whether new tsetse fly ecotypes might display different behaviour, dispersal patterns, host preferences and vectorial capacities. This work also urges a revision of taxonomic status of *Glossina palpalis* subspecies and highlights again how fast ecological divergence can be, especially in host-parasite-vector systems.

## Author Summary

The role of environmental factors in driving adaptive trajectories of living organisms is still being debated. This is even more important to understand when dealing with important and /or neglected diseases and their vectors. In this paper, we analysed genetic divergence, computed from several genetic markers, of 614 tsetse flies (*Glossina palpalis gambiensis* and *Glossina palpalis palpalis*, major vectors of animal and human trypanosomes) from 28 sites of West and Central Africa. We found that the two subspecies are so divergent that they deserve the species status. We found that *G*. *p*. *gambiensis* from different landscapes (Niayes of Senegal, savannah and coastal environments) were significantly genetically different, and thus represent different adaptive entities or even subspecies. We also confirm that *G*. *p*. *palpalis* from Ivory Coast, Cameroon and DRC are strongly divergent. These results provide an opportunity to examine whether these different types of tsetse fly might display different behaviour, dispersal patterns, host preferences and vectorial capacities. This work also urges a revision of taxonomic status of *Glossina palpalis* subspecies and highlights again how fast ecological divergence can be, especially in host-parasite-vector systems.

## Introduction

A capital step for species diversification is the initiation of some kind of disruptive selection, driving the newly diverged group of entities to some level of genetic adaptive divergence [1,2]. There has been a continuous debate on the respective role of geography and ecology in speciation, especially the speed at which these factors drive organisms to divergence [3]. These debates are important as they focus on key processes involved in evolution. For parasites and their vectors, the role of ecology and geography in driving divergence has important implications for management, as rapid evolution can occur in response to control practices or introductions to new environments [4]. This can have consequences on dispersal capacity [4], behaviour [5] and vectorial capacities [6–8].

Tsetse flies (Diptera: Glossinidae) are the sole cyclical vectors of human (HAT or sleeping sickness) and animal (AAT or nagana) African trypanosomoses, two major plagues that are seriously impeding African development [9]. Among these, *Glossina palpalis palpalis* and *Glossina palpalis gambiensis*, which are major vectors of both HAT and AAT, have recently been the subject of several population genetics studies (see [10] for a review). These studies, mainly based on spatio-temporal variation at microsatellite loci, have recurrently revealed some degree of genetic divergence, in some cases above the reasonable amount expected from geographically based population structure [11–14]. Because control programs against trypanosomoses often rely on tsetse eradication or suppression, it is important to specify the amount of such divergences and, if possible, if it could be linked to some ecological factors. Indeed, adaptive divergence may be correlated to variation in behaviour, host preference (or attractiveness to trapping devices) and vectoring ability.

In this paper, we combined and synthesized published and unpublished microsatellite data sets of these two taxa from populations sampled in West Africa and central Africa. We analysed the whole data set in order to evaluate the genetic divergence between the two taxa as assessed with microsatellite markers and then we analysed separately *G*. *p*. *gambiensis* and *G*. *p*. *palpalis* in order to assess the respective role of geographic distance, date of capture (time distance), landscape type and river basin in determining the level of genetic divergence of tsetse flies. The observed levels of divergence provide support for changes in the taxonomic status of these subspecies. Furthermore, based on both genetic and ecological criteria, we propose that several additional taxonomic groups should be recognized. The importance of these findings in developing novel control strategies and facilitating future research endeavours is discussed. Subspecies *G*. *p*. *gambiensis* and *G*. *p*. *palpalis* may have split no more than 13000 years ago [15,16]. The ecotypes evidenced in the present study necessarily are much younger and illustrate on how swift ecological divergence can be.

## Material and Methods

### Study sites

**Fig 1:**
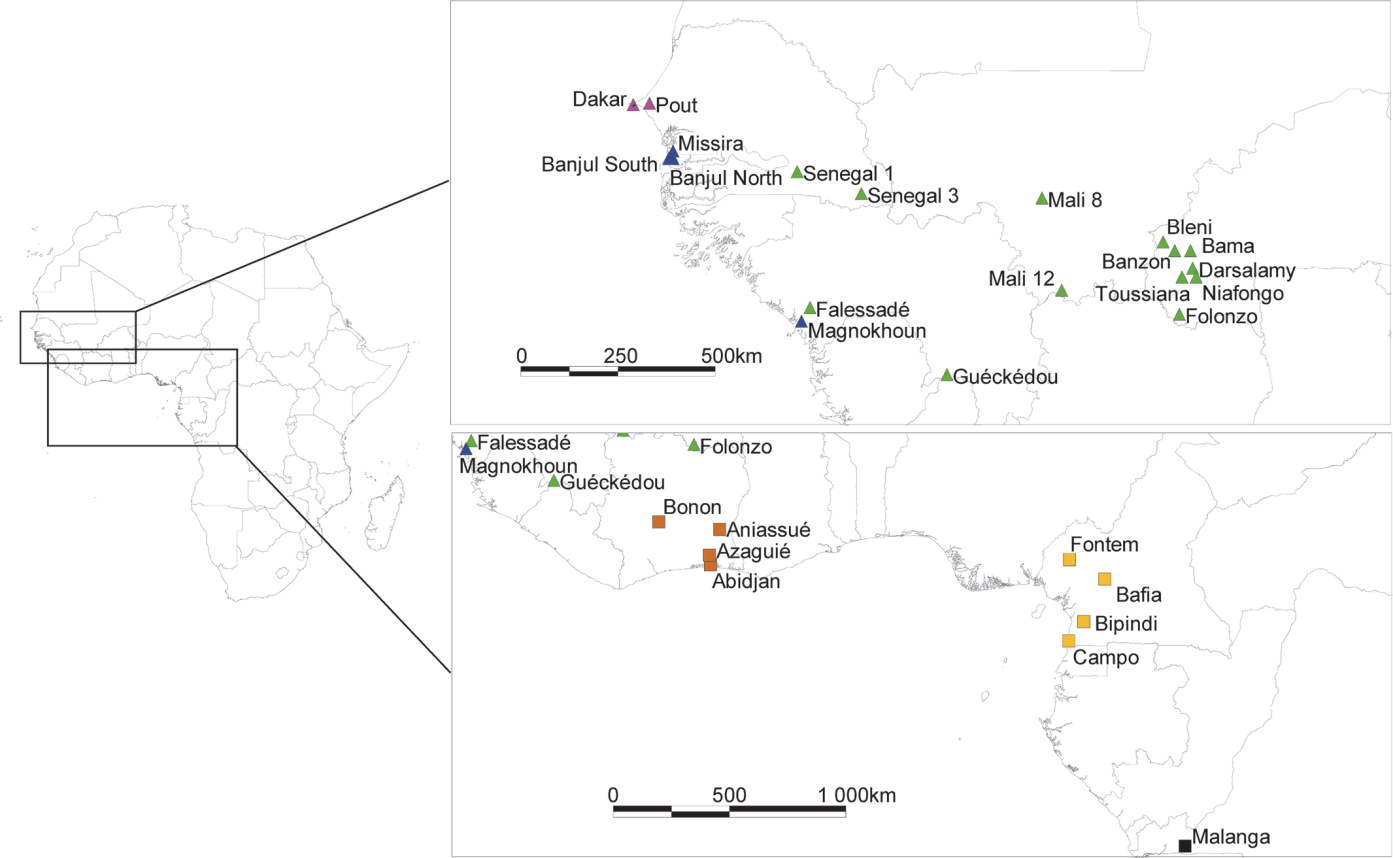
Geographic locations of sampled tsetse flies. *Glossina palpalis gambiensis* samples come from Dakar, Pout, Missira, S1 (Senegal 1) and S3 (Senegal 3) (Senegal); Banjul North and Banjul South (Banjul) (Gambia); Magnokhoun (Mag*), Falessadé, Bani and Guéckédou (Guinea); M8 (Mali 8) and M12 (Mali 12) (Mali); Bleni, Tou* (Toussiana), Bama, Ban* (Banzon), Dar* (Darsalamy), Nia* (Niafongo) and Folonzo (Burkina-Faso). *Glossina palpalis palpalis* samples come from Bonon, Aniassue, Azaguié and Abidjan (Ivory Coast); Fontem, Bafia Bipindi and Campo (Cameroon); and Malanga (Democratic Republic of Congo). * indicates abbreviated names. For *G*. *p*. *gambiensis* sites, Niayes sites are in purple, coastal sites in blue and savannah sites in green; for *G*. *p*. *palpalis* sites, Ivory-Coast sites are in brown, Cameroon in light brown and DRC in black (see also Fig. 2).

Study sites are located as represented in Fig. 1. The species, country, landscape type, river basin, date of capture, GPS coordinates and sample sizes are presented in Table 1. Raw data are available in S1 Table.

**Table 1:** Characteristics of subsamples of *Glossina palpalis gambiensis* (Gpg) and *Glossina palpalis palpalis* (Gpp) in different countries and different sites.

Species	Country	Site	Landscape	River Basin	Date	Lat	Long	*N*	Reference
Gpg	Burkina Faso	Bama	Savannah	Volta	11/07	11.4	-4.41	22	[17]
Gpg	Burkina Faso	Banzon	Savannah	Volta	03/08	11.4	-4.78	22	[9]
Gpg	Burkina Faso	Bleni	Savannah	Niger	03/08	11.6	-5.05	11	[9]
Gpg	Burkina Faso	Darsalamy	Savannah	Volta	03/08	11	-4.36	17	[9]
Gpg	Burkina Faso	Folonzo	Savannah	W. Coast	04/07	9.95	-4.67	12	Unpublished
Gpg	Burkina Faso	Niafongo	Savannah	Volta	03/08	10.8	-4.28	20	[9]
Gpg	Burkina Faso	Toussiana	Savannah	W. Coast	03/08	10.8	-4.61	6	[9]
Gpg	Gambia	Banjul North	Coast	W. Coast	07/06	13.5	-16.5	42	Unpublished
Gpg	Gambia	Banjul South	Coast	W. Coast	07/06	13.5	-16.6	9	Unpublished
Gpg	Guinea	Falessadé	Savannah	W. Coast	11/05	10.1	-13.3	16	[18]
Gpg	Guinea	Guéckédou	Savannah	W. Coast	05/07	8.57	-10.1	30	Unpublished
Gpg	Guinea	Magnokhoun	Coast	W. Coast	05/05	9.79	-13.5	21	[18]
Gpg	Mali	Mali 12	Savannah	Niger	03/09	10.5	-7.42	15	Unpublished
Gpg	Mali	Mali 8	Savannah	Niger	03/09	12.6	-7.88	20	Unpublished
Gpg	Senegal	Dakar	Niayes	W. Coast	07/07	14.73	-17.43	21	[12]
Gpg	Senegal	Missira	Coast	W. Coast	07/07	13.67	-16.50	22	[12]
Gpg	Senegal	Pout	Niayes	W. Coast	07/07	14.76	-17.05	3	[12]
Gpg	Senegal	Senegal 1	Savannah	W. Coast	02/08	13.2	-13.6	17	Unpublished
Gpg	Senegal	Senegal 3	Savannah	W. Coast	02/08	12.7	-12.1	17	Unpublished
Gpp	Cameroon	Bafia	Forest	Ctral W. Coast	10/09	4.75	11.24	18	[19]
Gpp	Cameroon	Bipindi	Forest	Ctral W. Coast	07/09	3.10	10.43	52	[19]
Gpp	Cameroon	Campo	Forest	Ctral W. Coast	04/09	2.35	9.85	51	[19]
Gpp	Cameroon	Fontem	Forest	Ctral W. Coast	04/09	5.50	9.88	24	[19]
Gpp	Democratic Republic of Congo	Malanga	Forest	Congo	08/09	-5.6	14.37	55	[19]
Gpp	Ivory Coast	Abidjan	Forest	W. Coast	10/07	5.30	-4.03	24	[61]
Gpp	Ivory Coast	Aniassué	Forest	W. Coast	04/07	6.66	-3.68	21	[61]
Gpp	Ivory Coast	Azaguié	Forest	W. Coast	03/11	5.67	-4.07	6	Unpublished
Gpp	Ivory Coast	Bonon	Forest	W. Coast	11/05	6.96	-6.04	20	[61]

The landscape type, river basin (W. designates West and Ctral designates Central), date of sampling (day/month), GPS coordinates in degrees decimal (Lat and Long in °C North and °C West), subsample sizes (*N*) and references are also given. River basins denominations follow the definitions from the FAO at http://www.fao.org/nr/water/aquastat/watresafrica/afr_basins.htm.

### Sampling

Most of the samples studied in this paper were already used and genotyped for publications relating to other, though related purposes. These papers are cited in Table 1 and sites samples can be seen in the Fig. 1. Folonzo sample was never published and was sampled during April 2007 following the same method as in [17]. Guekedou sample was never published and was sampled during May 2007 following the same procedure as in [18]; Senegal 1 and Senegal 3 samples were never published and were kindly provided by the Insect Pest Control Laboratory, Joint FAO/IAEA Program of Nuclear Techniques in Food and Agriculture and sampling followed the same procedures as described in [9]. Azaguié sample was never published and was sampled and genotyped for another project of our team by S. Ravel in collaboration with Dr D. Kaba (Pierre Richet / Institut National de Santé Publique, Abidjan, Ivory Coast) and Dr G. Acapovi-Yao (Laboratoire de Zoologie, Université d’Abidjan-Cocody, Abidjan, Ivory Coast) (Acapovi-Yao et al., manuscript in preparation). Published papers are available at http://gemi.mpl.ird.fr/SiteSGASS/SiteTDM/ArtiPDF.html. These 28 samples summed to 614 genotyped individuals, with 9 unpublished samples.

### Genotyping

Genotyping of unpublished data followed the same protocol as described in [17] and [19]. For Mali 12, Mali 8, Senegal 1 and Senegal 3, the genotypes of the flies were kindly provided by the Insect Pest Control Laboratory, Joint FAO/IAEA Program of Nuclear Techniques in Food and Agriculture and protocols were the same. Some of analyses undertaken do not tolerate missing data. For the sake of consistency between all analyses, only complete genotypes at seven loci were kept. These loci were: Gpg55.3 (X linked) [20]; B104 (X linked), B110 (X linked) and C102 that were kindly supplied by A. Robinson, Insect Pest Control Laboratory (formerly Entomology Unit), Food and Agricultural Organization of the United Nations/International Atomic Energy Agency [FAO/IAEA], Agriculture and Biotechnology Laboratories, Seibersdorf, Austria; pGp13 (X linked) and pGp24 [21]; and GPCAG [22]. Protocols followed what was described in references cited above (e.g. [18]). All genotyping were handled or supervised by the same person (SR) who ensured perfect calibration of allele sizes across sub-samples. A total of 614 tsetse flies from 28 sites displayed a full genotype at the seven microsatellite loci. All genotypic data were coded as they appeared, hence males were coded as homozygous at X-linked loci. Sex information was missing in samples from Gambia and assessed through genotypes found on X-linked loci.

### Data analyses

All data sets were built in appropriate text files and converted with Create V 1.1 [23] into the appropriate format as needed except for bootstrap analysis with Phylip for which we used Convert V 1.31 [24].

Genetic distances were computed with MSA 4.05 [25]. We used a Cavalli-Sforza and Edwards chord distance matrix [26] for dendrogram construction with a Neighbour-joining tree (NJTree) [27] and for regression analyses, as recommended [28,29]. The NJTree dendrogram showing relationships between all tsetse subsamples was built with Mega V 5 [30]. Robustness of nodes was assessed through 1000 bootstraps over loci with Phylip v 3.68 [31]. For that purpose, nodes in *G*. *p*. *gambiensis* were studied after rooting the tree with Malanga subsample (DRC), while for *G*. *p*. *palpalis* nodes, tree was rooted with Banjul North subsample (Gambia). Sample sizes are represented in Table 1.

Relationships between genetic distances and the other parameters were tested with partial Mantel tests (for robustness) and also explored with linear regressions (for illustrations and strength of signals measures). Explanatory variables and factors were the subspecies distance (whether the two compared samples contain the same subspecies or not), geographic distance (in km), time distance (in days), landscape and river basin (same or not). The different landscapes and river basins are presented in Table 1. For the Mantel tests, these factors were coded as 0 when the two sites compared shared the same value (e.g. both *G*. *p*. *gambiensis*) or 1 when different. For the linear regression, factors were coded as "Same" when similar in both sites and a combination of two modalities when different (e.g. Savannah-Coast). Mantel test for global data was undertaken to test for the effect of sub-speciation between *G*. *p*. *palpalis* and *G*. *p*. *gambiensis*. Because there are probably interactions with this effect, other factors were then analyzed more precisely within each subspecies separately. Partial Mantel tests were undertaken under Fstat V 2.9.3 [32] (updated from [33]) with 10000 Monte-Carlo randomizations of genetic distance matrix items. We also undertook Principal Component Analyses (PCA) on each sub-species data set. For this we used PCAGen 1.2.1 [34] that works on allele frequencies in subsamples and reorganize the data into a multidimensional space the metric of which is equivalent to Wright's *F*_ST_ [35], i.e. the part of inbreeding that is explained by population subdivision. The significance of the first axes was tested with the broken stick criterion [36] and also with 10000 permutations of individuals across subsamples. We then submitted subsample coordinates of each significant axis to multiple regressions. The general model to start with was always of the form Axis*i* ∼ Lat + Long + Day + Landscape + RiverBasin + Lat:Long where *i* identifies the axis number being investigated, Lat and Long mean the latitudinal and longitudinal GPS coordinates in degrees, Day means the number of days after the oldest sub-sample, Lanscape is as described above, RiverBasin is the name of the river basin as described above and ":" stands for interaction between two explanatory variables. Here the variables were weighted for subsample sizes.

All multiple regressions were undertaken under R [37] using sample sizes as weights. For all linear regressions, the best (minimum) model was selected after a stepwise procedure, using the Akaike Information Criterion [38], significance tested with a *F* test and multiple comparisons (when useful) were done with the Student-Newman-Keuls (SNK) test. Order of entry of explanatory variables matters both in Fstat (Mantel) and R analyses. We thus chose to enter these variables following an order of decreasing importance we thought they would have: geographic distance, time, landscape, basin and interactions (if any). Ecological factors were entered last to make sure the response was controlled for the other parameters.

Null alleles and X-linked loci produce an artificial excess of inbreeding in subsamples that should not affect the tests in any predictable direction but a decrease in power. These issues are thus relevant only in those cases where tests do not appear significant. A coming work involving one of the authors (TDM) will be devoted to the robustness of different genetic distances to such issues (manuscript in preparation).

NJTrees were also built without X-linked loci, on females only and on males only. This did not change the general aspect of the tree even if a few populations happened to branch in slightly different places. These NJTrees can be seen in S1 File.

### Data accessibility

The complete data set is available in S1 Table.

## Results

### Dendrogram

**Fig 2:**
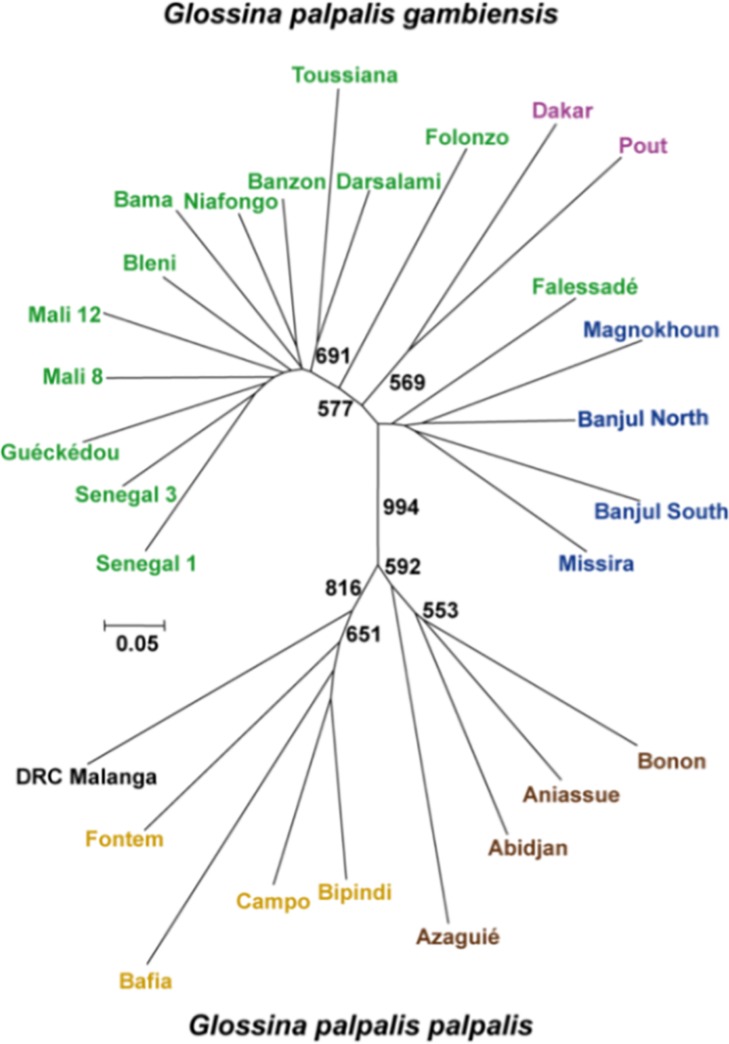
NJTree based on Cavalli-Sforza and Edwards chord distance between all subsamples of *G*. *palpalis* s.l. from West and Central Africa. *G*. *palpalis gambiensis* subsamples are in green (Savannah), purple (Niayes) and blue (Coast) respectively. *G*. *palpalis palpalis* subsamples are in brown (Ivory Coast), light brown (Cameroon) and black (DRC) respectively. Bootstrap values above 500 (out of 1000) are indicated. For geographic position see Fig. 1.

Geographic locations, landscape types and genetic relationships between all subsamples are presented in Table 1, Fig. 1 and Fig. 2. It can be seen that the two subspecies are clearly separated. In *G*. *p*. *gambiensis*, distinction between Savannah, Niayes and Coastal populations, in some instances, overcome geographic differentiation. This is particularly clear for samples from Gambia and Senegal (Fig. 1). For instance, as can be seen from Figs. 1 and 2, subsamples Senegal 1 and 3 are genetically closer to Burkina-Faso and Mali sites (Savannah) than from the geographically closer Dakar, Pout (Niayes), Missira, Banjul North and South (Coast). In *G*. *p*. *palpalis*, Central and Western African flies are clearly separated and geography seems to be the predominant factor within each of the two zones. Again, genetic distances are quite pronounced and bootstrap values relatively high.

### Partial Mantel, PCA and multiple regression approaches

Results of partial Mantel test for the whole data set provided a highly significant contribution of subspecies (partial *R* ^*2*^ = 0.65, *P*-value<0.0001).

**Fig 3:**
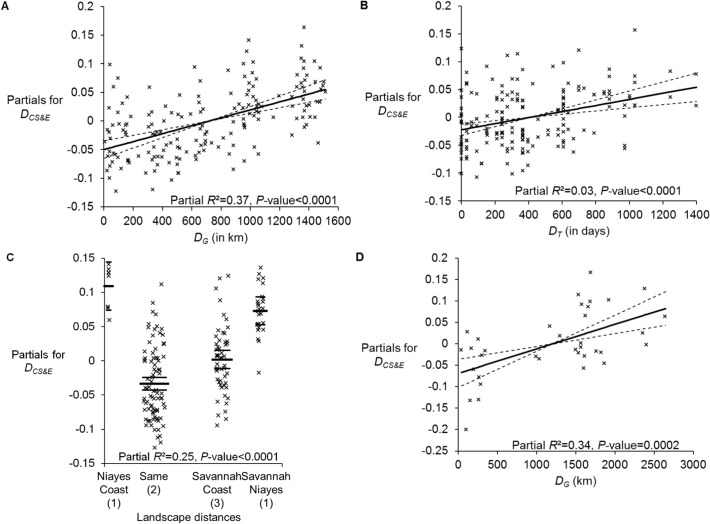
Results of multiple regressions between partials for genetic distances (*D*_*CS&E*_) and different parameters with 95% confidence intervals, percentage of total variance explained (*R* ^*2*^) and corresponding *P*-value (*F* test): *G*. *palpalis gambiensis* for geographic distance (3a), time distance (*D*_*T*_) (3b) and landscape distance (3c); *Glossina palpalis palpalis* for geographic distance (*D*_*G*_) (3d). For (3c), landscape comparisons with different letters are significantly different (SNK test). Statistics are given here only to illustrate the strength of the signals though the nature of the data does not allow using these formally.

For *G*. *p*. *gambiensis*, partial Mantel test highlighted two major factors that best explained genetic distance between subsamples (Table 2). The first is geographic distance, which explains 41% of the variance, followed by landscape distances that explain 10% of the variance of genetic distances and are highly significant. Other parameters (time and river basin) contribute little to the coefficient of determination *R* ^*2*^, though significantly so. Regarding the linear model, the stepwise procedure could not simplify the model. Nevertheless, river basin distances did not display consistent results since the response mainly was due to higher genetic distances between sites from the same basin as compared to other comparisons. This incoherence, which probably comes from interaction with geographic distance, led us to remove this factor from the analysis. Results are presented in Fig. 3. The total *R* ^*2*^ = 0.66. Here the main factor is geographic distance, followed by landscape distance. Both explained not less than 62% of the total genetic variance (which is quite big given the variation expected for genetic distances). Time explained very little of the variance though significant: the more time between subsamples, the more genetic divergence between them. For landscape distances, paired comparisons led to the conclusion that genetic distances between similar landscapes were smaller than any other comparison, and that Niayes subsamples were always genetically more distant from the other sites than any other comparison.

**Table 2:** Results of partial Mantel test for *Glossina palpalis gambiensis*.

Distances	Partial *R* ^*2*^	*P*-value
Geographic	0.41	0.0001
Time	0.04	0.0125
Landscape	0.10	0.0001
Basin	0.03	0.021
Total	0.57	0.0001

Partial determination coefficients (*R* ^*2*^, proportion of total variance explained) and corresponding *P*-values are presented

In *G*. *p*. *palpalis* subsamples, the Mantel test, partial *R* ^*2*^ and corresponding *P*-value are presented in Table 3. Only geographic distance displayed a significant effect here, with 34% of the variance explained. For the multiple regression approach, only geography seemed to play a significant role (partial *R* ^*2*^ = 0.34, *P*-value = 0.0002) (Fig. 3D). In particular, very high bootstrap values are observed between Central and West Africa and between Cameroon and RDC subsamples.

**Table 3:** Results of partial Mantel test for *Glossina palpalis palpalis*.

Distances	Partial *R* ^*2*^	*P*-value
Geographic	0.34	0.0002
Time	0.01	0.5308
Basin	0.01	0.5163
Total	0.37	0.0022

Partial determination coefficients (*R* ^*2*^, proportion of total variance explained) and corresponding *P*-values are presented. Only geographic distances have a significant effect.

For PCA analysis of *G*. *p*. *gambiensis* sub-samples, the first two axes appeared significant both with the broken stick criterion and with permutation testing (*P*-value≤0.0001 for Axis 1 and *P*-value = 0.0345 for Axis 2, permutation test). Axes 1 and 2 represent 41% and 16% of total inertia respectively. After stepwise procedures, Axis 1 is explained by all initial variables but Day (Table 4). By far the two most important variables are the latitude and the landscape that explain respectively 66% and 29% of the total variance in axis 1 (both *P*-values<0.0001). For the second axis, the minimum model was Axis2 ∼ Lat + Long + Landscape + RiverBasin (Table 5). Here, the most important variables are Lanscape and Latitude that respectively explain 39% and 30% of the total variance in axis 2 (*P*-values<0.00001).

**Table 4:** Minimum model obtained for the regression of PCA axis 1 coordinates of *Glossina palpalis gambiensis*.

Variables	Sum^*2*^	Partial *R* ^*2*^	*P*-value
Latitude	201.98	0.6639	0.0000
Longitude	3.07	0.0101	0.0039
Landscape	86.85	0.2855	0.0000
River basin	5.23	0.0172	0.0094
Lat:Long	2.42	0.0079	0.0088
Residuals	4.69		
*R* ^*2*^		0.9846	0.0000

The sum of squares (Sum^*2*^), the proportion of variance explained by the model (*R* ^*2*^) and the partial for each explanatory variable are given. The *P*-value was obtained after a *F* test

**Table 5:** Same as for Table 4 but for the second PCA axis of *Glossina palpalis gambiensis* subsamples.

Variable	Sum^*2*^	Partial *R* ^*2*^	*P*-value
Latitude	9.73	0.1396	0.0000
Longitude	20.96	0.3009	0.0000
Landscape	27.36	0.3928	0.0000
River basin	8.22	0.1180	0.0001
Residuals	3.40	0.0487	
*R* ^*2*^		0.9513	0.0000

For PCA analyses of *G*. *p*. *palpalis* sub-samples, the first three axes appeared significant both with the broken stick and permutation tests, with permutation *P*-value≤0.0001 for the two first axes and *P*-value = 0.011 for the third. They respectively represent 34, 27 and 17% of total inertia respectively. Here, variable Landscape was not introduced as it does not vary in the sampled zones for *G*. *p*. *palpalis*. For axis 1, no simplification of the model was possible (Table 6). The only significant effect comes from the latitude which explains 95% of the total variance on axis 1 (*P*-value = 0.0038). On axis 2, only two variables stayed in the model after the stepwise procedure (Table 7). However only longitude really mattered and explained no less than 94% of axis 2 (*P*-value≤0.0001). Finally, for axis 3, the minimum model was Axis3 ∼ Lat + Long + RiverBasin + Lat:Long and the most important explanatory variables were the river basin, explaining 81% of axis 3 variance (*P*-value = 0.0053), and the interaction between latitudinal and longitudinal coordinates that explained 14% of axis 3 variance (*P*-value = 0.0291) (Table 8).

**Table 6:** Results for the model obtained for the regression of PCA axis 1 coordinates of *Glossina palpalis palpalis*.

Variables	Sum^*2*^	Partial *R* ^*2*^	*P*-value
Latitude	37.03	0.9478	0.0038
Longitude	0.66	0.0169	0.1618
Day	0.11	0.0029	0.4635
River basin	0.91	0.0232	0.2356
Lat:Long	0.08	0.0019	0.5375
Residuals	0.28		
*R* ^*2*^		0.9928	

The sum of squares (Sum^*2*^), the proportion of variance explained by the model (*R* ^*2*^) and the partial for each explanatory variable are given. The *P*-value was obtained after a *F* test.

Lat:Long: interaction between latitude and longitude variables

**Table 7:** As for Table 6 but with axis 2 of *Glossina palpalis palpalis* sub-samples PCA coordinates.

Variables	Sum^*2*^	Partial *R* ^*2*^	*P*-value
Latitude	0.39	0.0123	0.2796
Longitude	29.49	0.9355	0.0000
Residuals	1.65		
*R* ^*2*^		0.9478	

**Table 8:** As for Table 7 but with axis 3 of *Glossina palpalis palpalis* sub-samples PCA coordinates.

Variables	Sum^*2*^	Partial *R* ^*2*^	*P*-value
Latitude	0.40	0.0204	0.2271
Longitude	0.00	0.0002	0.8814
River basin	15.74	0.8140	0.0057
Lat:Long	2.68	0.1386	0.0291
Residuals	0.52		
*R* ^*2*^		0.9733	

Lat:Long: interaction between latitude and longitude variables

## Discussion

The importance of geographic distance for determining genetic relationships between tsetse populations has been recurrently reported [9,19,39]. Its predominant effect above the effect of river basin was an expected result, at least for *G*. *p*. *gambiensis* [9] and is newly demonstrated here for *G*. *p*. *palpalis*.

The genetic distance that separates the two subspecies and the high bootstrap level obtained with microsatellite markers (known for their homoplasic nature) are advocating for a revision of the nomenclature of those taxa as different species. This is also in line with the biological definition of species, although the usefulness of such a concept is debatable [40], since the heterozygous males of the F1 crossing between these taxa are completely sterile [41,42] which leads to a very sharp allopatry between them in Ivory Coast [43,44]. Moreover, these taxa can be discriminated on a morphological basis using the size of the palette of the inferior claspers (larger in *G*. *p*. *gambiensis*) and the length of hairs on the inferior claspers (longer in *G*. *p*. *gambiensis*) [45]. This is even more justified as we also find evidence in the present paper of the existence of subunits within these two taxa, some of which are of an ecological nature.

The stronger impact of river basins on *G*. *p*. *gambiensis* than on *G*. *p*. *palpalis* is not surprising, taking into account that the savannah environment of the former makes it much more difficult to cross the interfluve than the dense forest environment of the latter. Time did not play a very pronounced effect on *G*. *p*. *gambiensis* and apparently had no effect on *G*. *p*. *palpalis*. For the latter, smaller sample sizes are probably the cause of this absence of detectable effect. For *G*. *p*. *gambiensis*, the significance of the effect is in line with genetic drift due to small effective population sizes that could be estimated in several studies in these taxa [9,10,12,17–19,39,46] but also in other tsetse taxa (see [47] for review). It highlights the need to take into account this factor in population genetics studies and the necessity to avoid pooling individuals that do not belong to the same cohort, in particular to estimate population differentiation, isolation by distance and migration.

In *G*. *p*. *gambiensis*, an important and significant effect of landscape where tsetse flies are found was evidenced. Interestingly, in several instances, genetic distances between subsamples from different landscapes are far above those between subsamples from the same landscape, even between the most remote ones. This strong impact of landscape was confirmed by the regression analyses where this variable explained as much, and sometimes more, the genetic composition of *G*. *p*. *gambiensis* sub-samples. Our study also confirms the genetic isolation of *G*. *p*. *gambiensis* from the Niayes [12,48] which has led to an eradication program in Senegal (http://www.fao.org/news/story/en/item/211898/icode/). It is clear from the different analyses that tsetse from the Niayes (Senegal) represent an objective subspecies, adapted to a specific environment [48,49]. This subspecies is able to reproduce in the complete absence of perennial hydrographic network. Moreover, tsetse from savannah and those from coastal landscapes also represent original diverged entities that can deserve the denomination of ecotypes, if not subspecies. There is however no pre- or post-mating barriers between these taxa, as evidenced by successful mating observed between tsetse flies from the Niayes and savannah tsetse flies from Mali and Burkina-Faso [50]. They can thus be considered as subspecies. It is the first time that such subspecies are evidenced. It has to be underlined that the discovery of these ecotypes may have important consequences. In particular, data from several studies made in the coastal part of Guinea have shown that the *G*. *p*. *gambiensis* ecotype caught in the sleeping sickness foci of this country do not display any infection with the pathogenic trypanosomes usually identified (including human and animal trypanosomes) in this species. Nevertheless, *G*. *p*. *gambiensis* is the only vector of sleeping sickness there [51,52]. To what extent the fact that they constitute a distinct ecotype can be linked to a different vector capacity remains to be documented, but may be of paramount importance for control programmes against both human and animal trypanosomoses. It was also demonstrated that *Trypanosoma brucei gambiense* from Guinea were genetically very different than those from Ivory Coast, and that this was probably due to the fact that they were not transmitted by the same tsetse taxa, i.e. *G*. *p*. *gambiensis* of the coastal landscape for *T*. *b*. *gambiense* from Guinea, and *G*. *p*. *palpalis* for the *T*. *b*. *gambiense* from Ivory Coast [53].

For *G*. *p*. *palpalis*, the very high bootstrap values observed between Central and West Africa and between Cameroon and RDC subsamples suggest subspeciation, if not more, in the ecological sense of it (adaptively divergent but not necessarily sexually isolated entities, see [40,54]). The existence of three subspecies (or even species) separating flies from West Africa (Ivory Coast), South of Cameroon, Equatorial Guinea and DRC has already been suggested, based on mtDNA (COI) [13] and there are probably more than that [55]. Here, our seven microsatellite loci provide a strong confirmation that *G*. *p*. *palpalis* is a strongly heterogeneous taxon. Moreover, [56] found significant differences in the morphology of the head between *G*. *p*. *palpalis* from West Africa and DRC. Regression analyses on PCA axes also highlighted the relevance of river basins. Nevertheless, many sites in the range of this species are missing (Gabon, Nigeria, Benin, Togo and Ghana) and other environmental measures are missing as well. Future studies, implying GIS approaches should bring more information and more precision on the mechanisms of ecotype and population delimitations in tsetse flies.

These observations are not only of academic interest as they have important repercussion as regard to vector control. Such ecological entities might represent different cases as regard to control success and reinvasion probabilities. It is thus key that such newly defined entities be ecologically characterized in order to compare their respective ecology (hygrometric and temperature preferences, host preferences, symbiotic flora and vector competences). Mating preferences or differential competitiveness may also alter the success of sterile insect technique (SIT) if inappropriate ecotypes are released in the wrong environment. This thus opens the gate to many and very productive new research topics on trypanosomes and their vectors. It also highlights how useful genetic markers can be in exploring the ecology of difficult organisms. Finally, our results call for an urgent taxonomic review of the status of *G*. *palpalis* subspecies. The split between *G*. *p*. *gambiensis* and *G*. *p*. *palpalis* was dated as old as 3.2 million years according to COI mtDNA assuming molecular clock [13]. Nevertheless, this result is based on a single mtDNA marker known to behave very oddly at the beginning of a split (for less than 1 million year the divergence can vary from 4 to 20%) [57]. Moreover, because of their lack of neutrality [58–60], mtDNA markers might not be ideal to estimate divergence time. We thus prefer relying on experts of the life history of tsetse flies who dated the split between the two sub-species around 13000 years ago (around 91000 tsetse fly generations) when the initial forest was separated into two isolated masses by drought [15,16]. It is probably the most parsimonious interpretation of tsetse flies history. The mean genetic distance between the two taxa is 0.65 (which is very high for a distance bonded to 1). It is 0.48 between savannah and coast subsamples, 0.53 between savannah and the Niayes and 0.5 between coast and the Niayes. Assuming constant microsatellite divergence with time, we can extrapolate that the ecological split in *G*. *palpalis gambiensis* occurred around 10000 years ago (around 70000 generations), hence at the end of last glaciation. These estimates probably correspond to considerable overestimates as divergence speed probably strongly decreased as the two sub-species increased in population size (which tends to freeze genetic drift) when meteorological constraints were progressively relaxed at the end of the Würm ice age. These results provide another powerful illustration on how swift ecological divergences can occur, in particular in host-parasite-vector systems [4,54].

## Supporting Information

S1 Table Raw data of *Glossina palpalis gambiensis* and *Glossina palpalis palpalis* with Species, Country, Site, Landscape, Date, Day, Bassin, FAO_hydroshed, Sex, SubSample and genotypes at loci X55_3, XpGp13, pGp24, XB104, XB110, C102 and GPCAG. (TXT)

S1 File NJTrees obtained with chord distance matrices on autosomal loci only, females only and males only. (PPTX)
