# Supplementary figures and images for: Ecotype Evolution in Glossina palpalis Subspecies, Major Vectors of Sleeping Sickness
Source: PLoS Negl Trop Dis. 2015 Mar 16;9(3):e0003497. doi: 10.1371/journal.pntd.0003497 (PMC4361538; doi:10.1371/journal.pntd.0003497)

## Slide 1
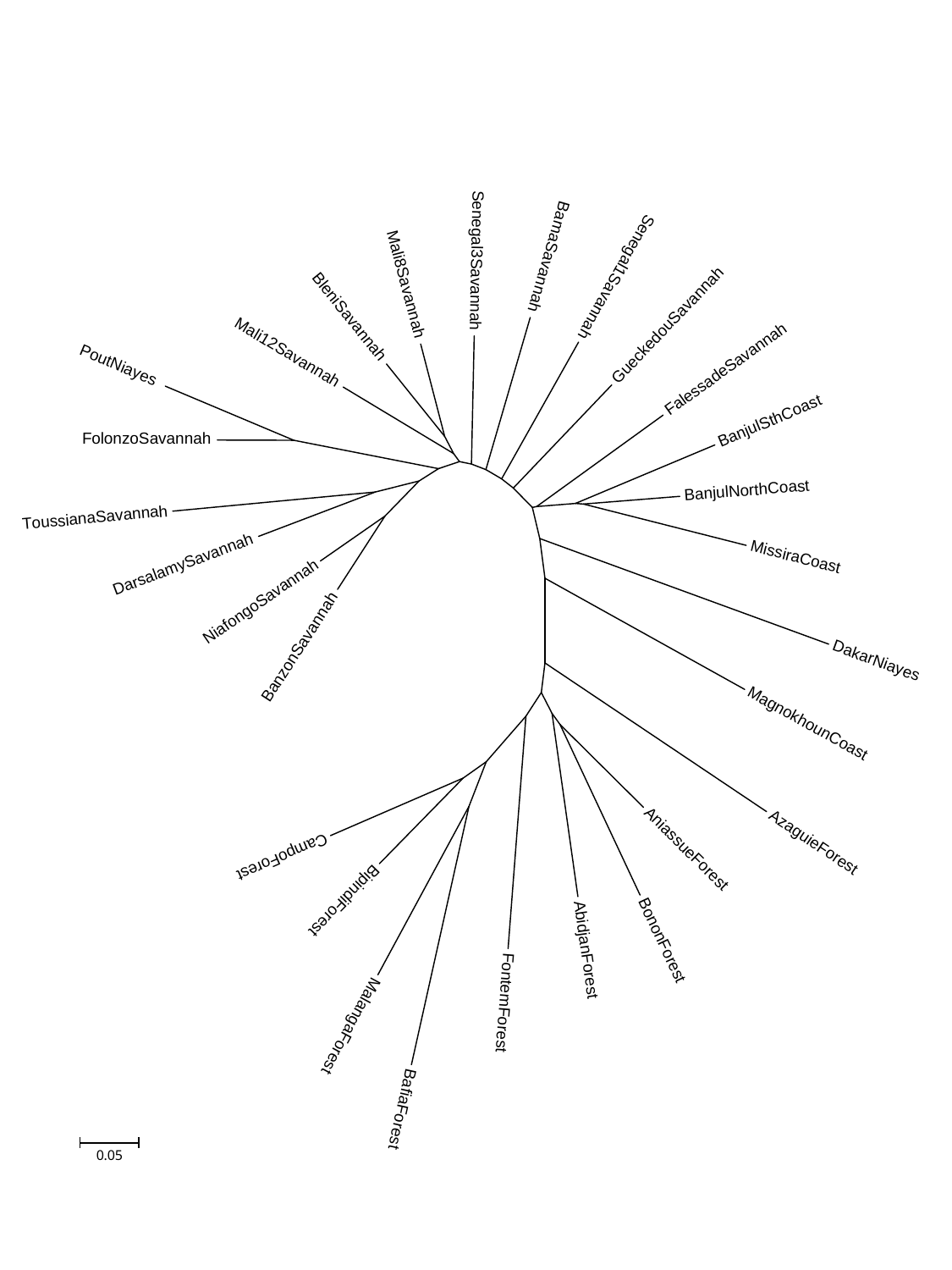

## Slide 2
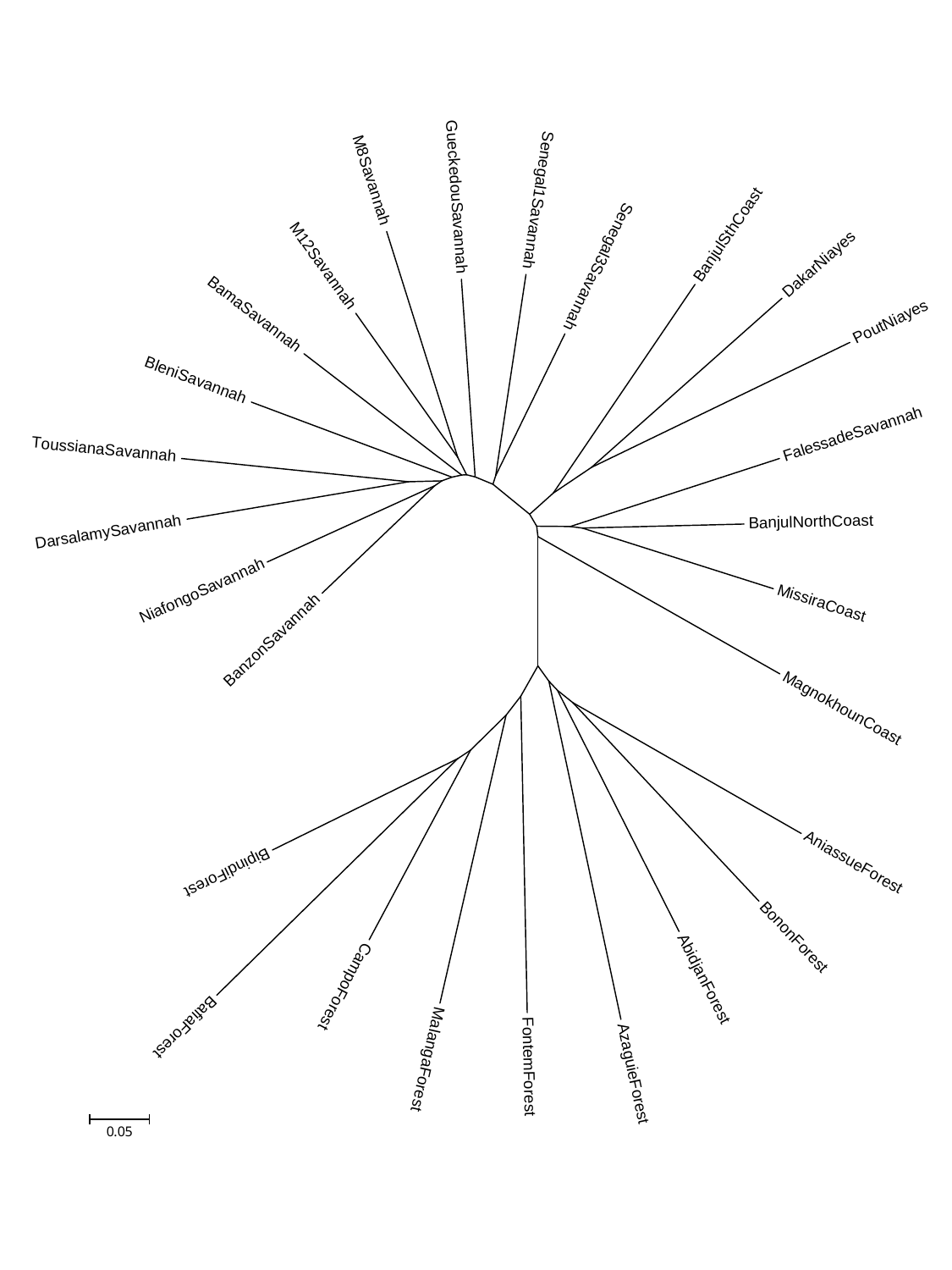

## Slide 3
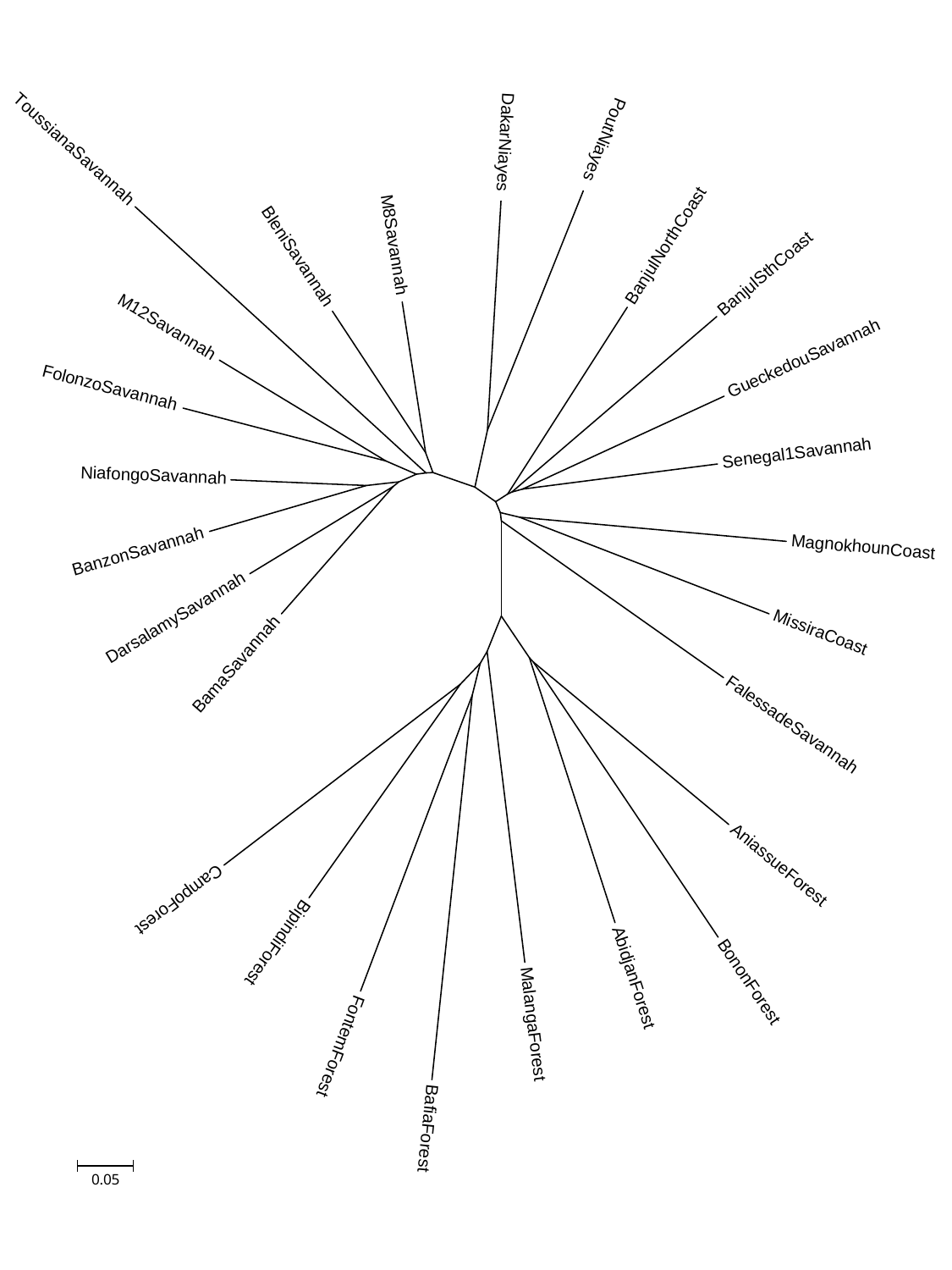

Supplement: S1 File — (PPTX) [file pntd.0003497.s002.pptx]
